# Crystal structure of [(2*S*,3*R*)-3-hy­droxy-3-phenyl­butan-2-yl]pyrrolidinium chloride

**DOI:** 10.1107/S2056989015016916

**Published:** 2015-09-17

**Authors:** Abirami Kandhaswamy, K.S. Meena, S. Deepa, S. Murugavel

**Affiliations:** a Department of Chemistry, Queen Mary’s College, Chennai 600 004, Tamilnadu, India; b Department of Physics, Government College of Engineering, Salem 636 011, India; c Department of Physics, Thanthai Periyar Government Institute of Technology, Vellore 632 002, India

**Keywords:** crystal structure, salt, pyrrolidinium chloride, ionic liquids, hydrogen bonding

## Abstract

In the title mol­ecular salt, C_14_H_22_NO^+^·Cl^−^, the pyrrolidinium ring adopts a twisted conformation about one of the N—C bonds. It is oriented at a dihedral angle of 42.0 (1)° with respect to the benzene ring. The torsion angle for the central N—C—C—C_ar_ (ar = aromatic) linkage is 163.74 (15)°. In the crystal, the components are linked *via* N—H⋯Cl and O—H⋯Cl hydrogen bonds, forming zigzig chains along the *b*-axis direction. These chains are connected along the *c* axis by very weak C—H⋯π inter­actions, forming a two-dimensional supra­molecular network.

## Related literature   

For background to pyrrolidinium-based ionic liquids, see: Henderson *et al.* (2006 ▸).
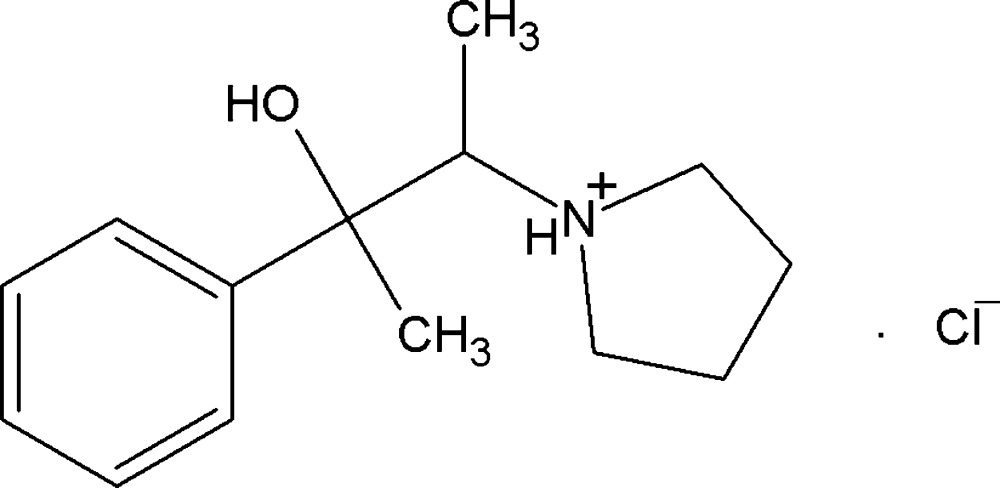


## Experimental   

### Crystal data  

C_14_H_22_NO^+^·Cl^−^
*M* *_r_* = 255.78
Orthorhombic, 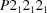

*a* = 7.3912 (14) Å
*b* = 9.7002 (17) Å
*c* = 19.727 (4) Å
*V* = 1414.4 (4) Å^3^
*Z* = 4
Mo *K*α radiation
μ = 0.26 mm^−1^
*T* = 293 K
0.24 × 0.21 × 0.16 mm

### Data collection  

Bruker SMART CCD diffractometer
Absorption correction: multi-scan (*SADABS*; Sheldrick, 1996 ▸) *T*_min_ = 0.940, *T*_max_ = 0.960
6666 measured reflections
3163 independent reflections
2750 reflections with *I* > 2σ(*I*)
*R*_int_ = 0.024

### Refinement  

*R*[*F* ^2^ > 2σ(*F* ^2^)] = 0.042
*wR*(*F* ^2^) = 0.107
*S* = 1.02
3163 reflections
160 parameters
H atoms treated by a mixture of independent and constrained refinement
Δρ_max_ = 0.19 e Å^−3^
Δρ_min_ = −0.17 e Å^−3^
Absolute structure: Flack (1983 ▸), 1235 Friedel pairs
Absolute structure parameter: −0.01 (7)

### 

Data collection: *SMART* (Bruker, 2002 ▸); cell refinement: *SAINT* (Bruker, 2002 ▸); data reduction: *SAINT*; program(s) used to solve structure: *SHELXS97* (Sheldrick, 2008 ▸); program(s) used to refine structure: *SHELXL97* (Sheldrick, 2008 ▸); molecular graphics: *ORTEP-3 for Windows* (Farrugia, 2012 ▸); software used to prepare material for publication: *SHELXL97* and *PLATON* (Spek, 2009 ▸).

## Supplementary Material

Crystal structure: contains datablock(s) global, I. DOI: 10.1107/S2056989015016916/hb7497sup1.cif

Structure factors: contains datablock(s) I. DOI: 10.1107/S2056989015016916/hb7497Isup2.hkl

Supporting information file. DOI: 10.1107/S2056989015016916/hb7497Isup3.cml

**Fig. 1.**
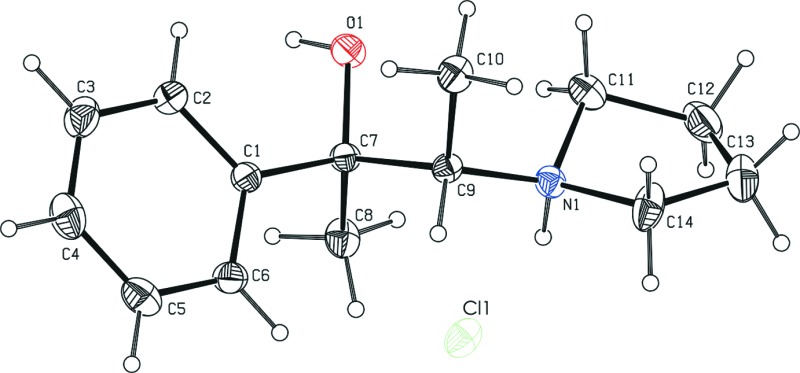
Molecular structure of the title compound showing displacement ellipsoids at the 30% probability level.

. DOI: 10.1107/S2056989015016916/hb7497fig1.tif
Mol­ecular structure of the title compound showing displacement ellipsoids at the 30% probability level.

**Fig. 2.**
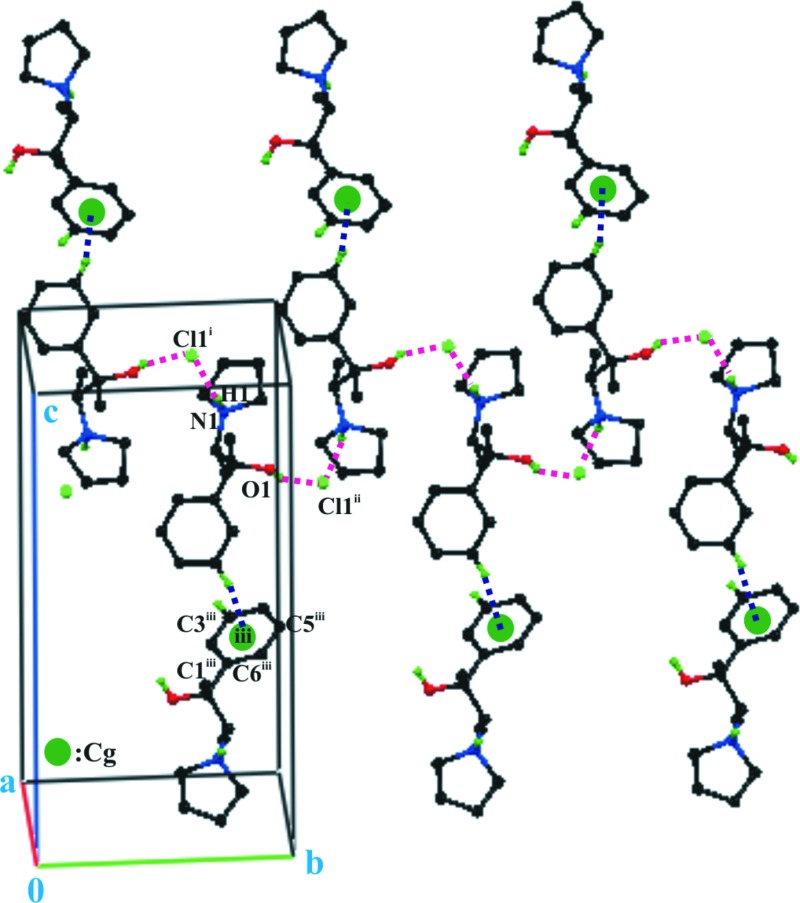
Part of the crystal structure showing intermolecular N—H···Cl, O—H···Cl and C—H···π interactions, forming a two dimensional supramolecular network. Hydrogen atoms not involved in hydrogen bonding are omitted for clarity. Cg is the centroid of C1–C6 benzene ring.

Cg . DOI: 10.1107/S2056989015016916/hb7497fig2.tif
Part of the crystal structure showing inter­molecular N—H⋯Cl, O—H⋯Cl and C—H⋯π inter­actions, forming a two dimensional supra­molecular network. Hydrogen atoms not involved in hydrogen bonding are omitted for clarity. *Cg* is the centroid of C1–C6 benzene ring.

CCDC reference: 1423292

Additional supporting information:  crystallographic information; 3D view; checkCIF report

## Figures and Tables

**Table 1:** Hydrogen-bond geometry (, ) *Cg* is the centroid of the C1C6 benzene ring.

*D*H*A*	*D*H	H*A*	*D* *A*	*D*H*A*
N1H1Cl1^i^	0.89(2)	2.24(2)	3.0804(19)	158.0(2)
O1H1*A*Cl1^ii^	0.82	2.28	3.0456(14)	156
C3H3*Cg* ^iii^	0.93	2.93	3.630(3)	133

Symmetry codes: (i) 
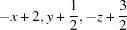
; (ii) 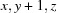; (iii) 
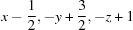
.

## supplementary crystallographic information

### S1. Chemical context

Pyrrolidinium-based ionic liquids have been a subject of intense investigation recently (Henderson *et al.*, 2006), whereby with understanding of the fundamental molecular-level inter­actions, a desired product with predicted physico-chemical properites could be designed. Additionally, a particular emphasis has been placed on whether hydrogen bonding occurs between the cation and a potential electron-pair donor (hydrogen bond acceptor) and its influence on the ionic liquids' overall properties. Against this background, and in order to obtain detailed information on molecular conformations in the solid state, X-ray studies of the title compound (I) have been carried out.

### S2. Structural commentary

Fig. 1. shows a displacement ellipsoid plot of (I), with the atom numbering scheme. In the cation, the pyrrolidinium ring adopts a twist conformation, with twist about the N1—C11 bond; the puckering parameters, q_2_ = 0.361 (2) Å and φ_2_ = 202.4 (5)°, and asymmetry parameters ΔC_2_[N1—C11] = 4.8.0 (3) Å. The pyrrolidinium ring is oriented at an angle 42.0 (1)° from the mean plane of the benzene ring.

### S3. Supra­molecular features

In the crystal, cations and anions are linked via N1—H1···Cl1 and O1—H1A···Cl1 hydrogen bonds, forming a one-dimensional zig zig chain along the b-axis direction. These chains are stacked along the c-axis by C—H···π inter­actions, between benzene H atom and the benzene ring of an adjacene molecule, with a C3—H3···Cg^iii^, forming a two-dimensional supra­molecular network. (Table 1 and Fig. 2; Cg is centroid of C1–C6 benzene ring. Symmetry code: (iii) -1/2+x,3/2-y,1-z>).

### S4. Synthesis and crystallization

In a round bottomed flask, a stirred solution of (S)-pyrrolidinylnorephedrone was reacted with methyl­magnesium bromide in THF medium over a period of 3 h. The completion of the reaction was monitored by thin layer chromatographic analysis. Further the crude mass was quenched with dil HCl and extracted with ethyl acetate under reduced vacuum. The resulting compound was recrystallised from EtOH/H_2_O (4:1) solution as colourless blocks in good yield (86%).

### S5. Refinement

Crystal data, data collection and structure refinement details are summarized in Table 2. N-bound H atom was located in a difference Fourier map and freely refined. All other H atoms were positioned geometrically and constrained to ride on their parent atom with C—H = 0.93–0.97 Å and with *U*_iso_(H)=1.5*U*_eq_ for methyl H atoms and 1.2*U*_eq_(C) for other H atoms. Owing to poor agreement, the reflection (-3 2 4) was omitted from the final cycles of refinement.

### Crystal data

**Table:**

C_14_H_22_NO^+^·Cl^−^	*F*(000) = 552
*M**_r_* = 255.78	*D*_x_ = 1.201 Mg m^−^^3^
Orthorhombic, *P*2_1_2_1_2_1_	Mo *K*α radiation, λ = 0.71073 Å
Hall symbol: P 2ac 2ab	Cell parameters from 3836 reflections
*a* = 7.3912 (14) Å	θ = 3.7–29.2°
*b* = 9.7002 (17) Å	µ = 0.26 mm^−^^1^
*c* = 19.727 (4) Å	*T* = 293 K
*V* = 1414.4 (4) Å^3^	Block, colourless
*Z* = 4	0.24 × 0.21 × 0.16 mm

### Data collection

**Table:**

Bruker SMART CCD diffractometer	3163 independent reflections
Radiation source: fine-focus sealed tube	2750 reflections with *I* > 2σ(*I*)
Graphite monochromator	*R*_int_ = 0.024
φ and ω scans	θ_max_ = 29.2°, θ_min_ = 3.7°
Absorption correction: multi-scan (*SADABS*; Sheldrick, 1996)	*h* = −9→8
*T*_min_ = 0.940, *T*_max_ = 0.960	*k* = −12→12
6666 measured reflections	*l* = −25→26

### Refinement

**Table:**

Refinement on *F*^2^	Secondary atom site location: difference Fourier map
Least-squares matrix: full	Hydrogen site location: inferred from neighbouring sites
*R*[*F*^2^ > 2σ(*F*^2^)] = 0.042	H atoms treated by a mixture of independent and constrained refinement
*wR*(*F*^2^) = 0.107	*w* = 1/[σ^2^(*F*_o_^2^) + (0.0569*P*)^2^ + 0.1443*P*] where *P* = (*F*_o_^2^ + 2*F*_c_^2^)/3
*S* = 1.02	(Δ/σ)_max_ < 0.001
3163 reflections	Δρ_max_ = 0.19 e Å^−^^3^
160 parameters	Δρ_min_ = −0.16 e Å^−^^3^
0 restraints	Absolute structure: Flack (1983), 1235 Friedel pairs
Primary atom site location: structure-invariant direct methods	Absolute structure parameter: −0.01 (7)

### Special details

**Table:**

Geometry. All esds (except the esd in the dihedral angle between two l.s. planes) are estimated using the full covariance matrix. The cell esds are taken into account individually in the estimation of esds in distances, angles and torsion angles; correlations between esds in cell parameters are only used when they are defined by crystal symmetry. An approximate (isotropic) treatment of cell esds is used for estimating esds involving l.s. planes.
Refinement. Refinement of F^2^ against ALL reflections. The weighted R-factor wR and goodness of fit S are based on F^2^, conventional R-factors R are based on F, with F set to zero for negative F^2^. The threshold expression of F^2^ > 2sigma(F^2^) is used only for calculating R-factors(gt) etc. and is not relevant to the choice of reflections for refinement. R-factors based on F^2^ are statistically about twice as large as those based on F, and R- factors based on ALL data will be even larger.

### Fractional atomic coordinates and isotropic or equivalent isotropic displacement parameters (Å^2^)

**Table:**

	*x*	*y*	*z*	*U*_iso_*/*U*_eq_	
C1	0.7420 (2)	0.74116 (19)	0.62918 (9)	0.0356 (4)	
C2	0.6667 (3)	0.8068 (2)	0.57376 (10)	0.0478 (5)	
H2	0.6323	0.8988	0.5772	0.057*	
C3	0.6418 (4)	0.7369 (3)	0.51298 (12)	0.0596 (6)	
H3	0.5911	0.7826	0.4762	0.071*	
C4	0.6913 (4)	0.6009 (3)	0.50673 (12)	0.0623 (6)	
H4	0.6728	0.5542	0.4661	0.075*	
C5	0.7685 (3)	0.5345 (2)	0.56109 (13)	0.0568 (6)	
H5	0.8043	0.4430	0.5570	0.068*	
C6	0.7931 (3)	0.6034 (2)	0.62179 (11)	0.0440 (4)	
H6	0.8446	0.5571	0.6583	0.053*	
C7	0.7703 (2)	0.81613 (18)	0.69701 (9)	0.0345 (4)	
C8	0.9719 (3)	0.8127 (3)	0.71443 (10)	0.0490 (5)	
H8A	1.0408	0.8444	0.6762	0.074*	
H8B	1.0069	0.7200	0.7253	0.074*	
H8C	0.9946	0.8715	0.7527	0.074*	
C9	0.6487 (3)	0.74346 (18)	0.75061 (9)	0.0333 (4)	
H9	0.6670	0.6440	0.7451	0.040*	
C10	0.4494 (3)	0.7716 (2)	0.73699 (10)	0.0461 (5)	
H10A	0.3768	0.7182	0.7676	0.069*	
H10B	0.4209	0.7464	0.6912	0.069*	
H10C	0.4248	0.8678	0.7436	0.069*	
C11	0.6819 (3)	0.9254 (2)	0.84700 (11)	0.0505 (5)	
H11A	0.7778	0.9828	0.8289	0.061*	
H11B	0.5659	0.9641	0.8341	0.061*	
C12	0.6971 (4)	0.9122 (3)	0.92291 (12)	0.0723 (8)	
H12A	0.8227	0.9160	0.9370	0.087*	
H12B	0.6310	0.9855	0.9454	0.087*	
C13	0.6170 (6)	0.7756 (3)	0.93918 (14)	0.0910 (11)	
H13A	0.6936	0.7271	0.9712	0.109*	
H13B	0.4988	0.7878	0.9597	0.109*	
C14	0.6001 (3)	0.6945 (3)	0.87578 (10)	0.0612 (6)	
H14A	0.4741	0.6830	0.8633	0.073*	
H14B	0.6547	0.6041	0.8810	0.073*	
N1	0.7001 (2)	0.77830 (17)	0.82284 (7)	0.0369 (4)	
O1	0.7091 (2)	0.95487 (12)	0.69491 (7)	0.0457 (4)	
H1A	0.7648	0.9970	0.6655	0.069*	
Cl1	0.93476 (8)	0.17271 (6)	0.62270 (3)	0.05642 (18)	
H1	0.817 (3)	0.761 (2)	0.8290 (9)	0.034 (5)*	

### Atomic displacement parameters (Å^2^)

**Table:**

	*U*^11^	*U*^22^	*U*^33^	*U*^12^	*U*^13^	*U*^23^
C1	0.0296 (9)	0.0393 (9)	0.0378 (9)	0.0004 (7)	0.0036 (7)	0.0035 (7)
C2	0.0494 (12)	0.0502 (12)	0.0439 (10)	0.0079 (10)	−0.0003 (9)	0.0068 (9)
C3	0.0524 (14)	0.0828 (16)	0.0434 (12)	0.0025 (13)	−0.0078 (10)	0.0065 (11)
C4	0.0620 (16)	0.0770 (16)	0.0479 (13)	−0.0072 (14)	0.0016 (11)	−0.0190 (11)
C5	0.0570 (15)	0.0475 (12)	0.0660 (15)	0.0006 (11)	0.0101 (11)	−0.0127 (10)
C6	0.0413 (10)	0.0425 (10)	0.0483 (11)	0.0046 (8)	0.0023 (9)	0.0038 (9)
C7	0.0318 (9)	0.0340 (8)	0.0378 (9)	0.0009 (8)	0.0036 (7)	0.0037 (7)
C8	0.0312 (10)	0.0675 (14)	0.0485 (11)	−0.0083 (10)	0.0034 (8)	0.0011 (10)
C9	0.0328 (9)	0.0311 (8)	0.0360 (9)	−0.0024 (7)	0.0002 (7)	0.0031 (7)
C10	0.0324 (10)	0.0557 (12)	0.0503 (11)	−0.0086 (9)	0.0016 (9)	0.0070 (9)
C11	0.0484 (13)	0.0465 (11)	0.0566 (12)	0.0007 (10)	0.0044 (10)	−0.0112 (9)
C12	0.0666 (17)	0.095 (2)	0.0550 (14)	0.0253 (16)	−0.0025 (13)	−0.0263 (14)
C13	0.133 (3)	0.092 (2)	0.0482 (14)	0.025 (2)	0.0297 (16)	0.0091 (13)
C14	0.0536 (14)	0.0890 (18)	0.0411 (11)	−0.0199 (12)	0.0026 (10)	0.0173 (12)
N1	0.0311 (9)	0.0417 (8)	0.0379 (8)	−0.0014 (7)	0.0042 (7)	0.0009 (6)
O1	0.0526 (9)	0.0307 (6)	0.0538 (8)	−0.0013 (6)	0.0164 (7)	0.0050 (6)
Cl1	0.0548 (3)	0.0642 (3)	0.0503 (3)	−0.0250 (3)	−0.0023 (3)	0.0119 (2)

### Geometric parameters (Å, º)

**Table:**

C1—C2	1.382 (3)	C9—H9	0.9800
C1—C6	1.396 (3)	C10—H10A	0.9600
C1—C7	1.537 (3)	C10—H10B	0.9600
C2—C3	1.390 (3)	C10—H10C	0.9600
C2—H2	0.9300	C11—C12	1.507 (3)
C3—C4	1.375 (4)	C11—N1	1.511 (3)
C3—H3	0.9300	C11—H11A	0.9700
C4—C5	1.375 (4)	C11—H11B	0.9700
C4—H4	0.9300	C12—C13	1.486 (4)
C5—C6	1.383 (3)	C12—H12A	0.9700
C5—H5	0.9300	C12—H12B	0.9700
C6—H6	0.9300	C13—C14	1.483 (4)
C7—O1	1.420 (2)	C13—H13A	0.9700
C7—C8	1.530 (3)	C13—H13B	0.9700
C7—C9	1.557 (2)	C14—N1	1.516 (3)
C8—H8A	0.9600	C14—H14A	0.9700
C8—H8B	0.9600	C14—H14B	0.9700
C8—H8C	0.9600	N1—H1	0.89 (2)
C9—N1	1.513 (2)	O1—H1A	0.8200
C9—C10	1.522 (3)		
			
C2—C1—C6	117.9 (2)	C9—C10—H10B	109.5
C2—C1—C7	121.70 (17)	H10A—C10—H10B	109.5
C6—C1—C7	120.44 (18)	C9—C10—H10C	109.5
C1—C2—C3	120.7 (2)	H10A—C10—H10C	109.5
C1—C2—H2	119.6	H10B—C10—H10C	109.5
C3—C2—H2	119.6	C12—C11—N1	103.1 (2)
C4—C3—C2	120.7 (2)	C12—C11—H11A	111.2
C4—C3—H3	119.6	N1—C11—H11A	111.2
C2—C3—H3	119.6	C12—C11—H11B	111.2
C3—C4—C5	119.3 (2)	N1—C11—H11B	111.2
C3—C4—H4	120.3	H11A—C11—H11B	109.1
C5—C4—H4	120.3	C13—C12—C11	105.1 (2)
C4—C5—C6	120.3 (2)	C13—C12—H12A	110.7
C4—C5—H5	119.9	C11—C12—H12A	110.7
C6—C5—H5	119.9	C13—C12—H12B	110.7
C5—C6—C1	121.1 (2)	C11—C12—H12B	110.7
C5—C6—H6	119.4	H12A—C12—H12B	108.8
C1—C6—H6	119.4	C14—C13—C12	108.9 (2)
O1—C7—C8	109.73 (17)	C14—C13—H13A	109.9
O1—C7—C1	112.29 (15)	C12—C13—H13A	109.9
C8—C7—C1	108.54 (16)	C14—C13—H13B	109.9
O1—C7—C9	105.37 (14)	C12—C13—H13B	109.9
C8—C7—C9	113.58 (16)	H13A—C13—H13B	108.3
C1—C7—C9	107.36 (15)	C13—C14—N1	104.8 (2)
C7—C8—H8A	109.5	C13—C14—H14A	110.8
C7—C8—H8B	109.5	N1—C14—H14A	110.8
H8A—C8—H8B	109.5	C13—C14—H14B	110.8
C7—C8—H8C	109.5	N1—C14—H14B	110.8
H8A—C8—H8C	109.5	H14A—C14—H14B	108.9
H8B—C8—H8C	109.5	C11—N1—C9	119.07 (15)
N1—C9—C10	111.66 (16)	C11—N1—C14	104.25 (17)
N1—C9—C7	113.19 (15)	C9—N1—C14	114.00 (15)
C10—C9—C7	110.96 (15)	C11—N1—H1	102.7 (13)
N1—C9—H9	106.9	C9—N1—H1	109.3 (12)
C10—C9—H9	106.9	C14—N1—H1	106.2 (13)
C7—C9—H9	106.9	C7—O1—H1A	109.5
C9—C10—H10A	109.5		
			
C6—C1—C2—C3	0.5 (3)	C1—C7—C9—N1	163.74 (15)
C7—C1—C2—C3	−179.8 (2)	O1—C7—C9—C10	50.1 (2)
C1—C2—C3—C4	0.1 (4)	C8—C7—C9—C10	170.19 (18)
C2—C3—C4—C5	−0.9 (4)	C1—C7—C9—C10	−69.8 (2)
C3—C4—C5—C6	1.1 (4)	N1—C11—C12—C13	−32.0 (3)
C4—C5—C6—C1	−0.5 (3)	C11—C12—C13—C14	14.7 (3)
C2—C1—C6—C5	−0.3 (3)	C12—C13—C14—N1	8.7 (3)
C7—C1—C6—C5	180.00 (19)	C12—C11—N1—C9	165.87 (19)
C2—C1—C7—O1	0.7 (2)	C12—C11—N1—C14	37.5 (2)
C6—C1—C7—O1	−179.62 (17)	C10—C9—N1—C11	−62.2 (2)
C2—C1—C7—C8	−120.8 (2)	C7—C9—N1—C11	63.9 (2)
C6—C1—C7—C8	58.9 (2)	C10—C9—N1—C14	61.6 (2)
C2—C1—C7—C9	116.06 (19)	C7—C9—N1—C14	−172.33 (17)
C6—C1—C7—C9	−64.3 (2)	C13—C14—N1—C11	−28.6 (3)
O1—C7—C9—N1	−76.39 (18)	C13—C14—N1—C9	−160.0 (2)
C8—C7—C9—N1	43.7 (2)		

### Hydrogen-bond geometry (Å, º)

Cg is the centroid of the C1–C6 benzene ring.

**Table:**

*D*—H···*A*	*D*—H	H···*A*	*D*···*A*	*D*—H···*A*
N1—H1···Cl1^i^	0.89 (2)	2.24 (2)	3.0804 (19)	158.0 (2)
O1—H1*A*···Cl1^ii^	0.82	2.28	3.0456 (14)	156
C3—H3···*Cg*^iii^	0.93	2.93	3.630 (3)	133

Symmetry codes: (i) −*x*+2, *y*+1/2, −*z*+3/2; (ii) *x*, *y*+1, *z*; (iii) *x*−1/2, −*y*+3/2, −*z*+1.
