# Supplementary figures and images for: Crystal structure of [(2S,3R)-3-hy­droxy-3-phenyl­butan-2-yl]pyrrolidinium chloride
Source: Acta Crystallogr E Crystallogr Commun. 2015 Sep 17;71(Pt 10):o758. doi: 10.1107/S2056989015016916 (PMC4647393; doi:10.1107/S2056989015016916)

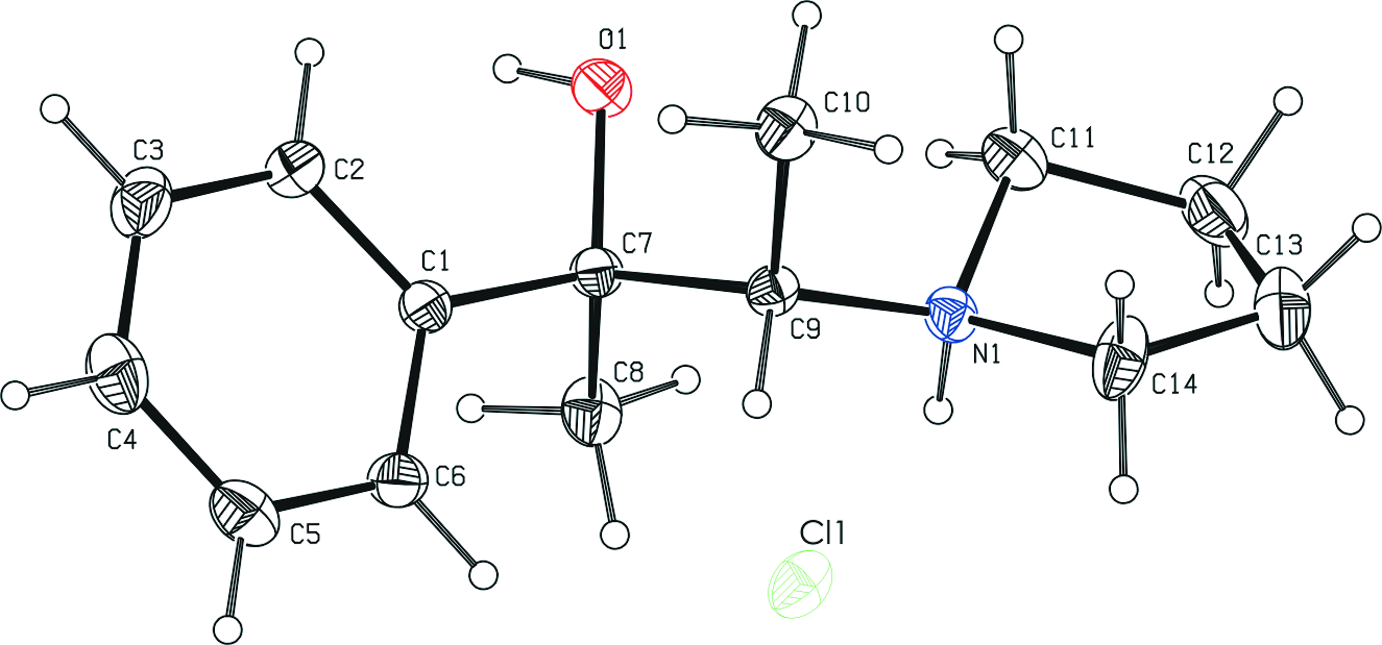

Supplement: Supplementary file 4 [file e-71-0o758-fig1.tif]

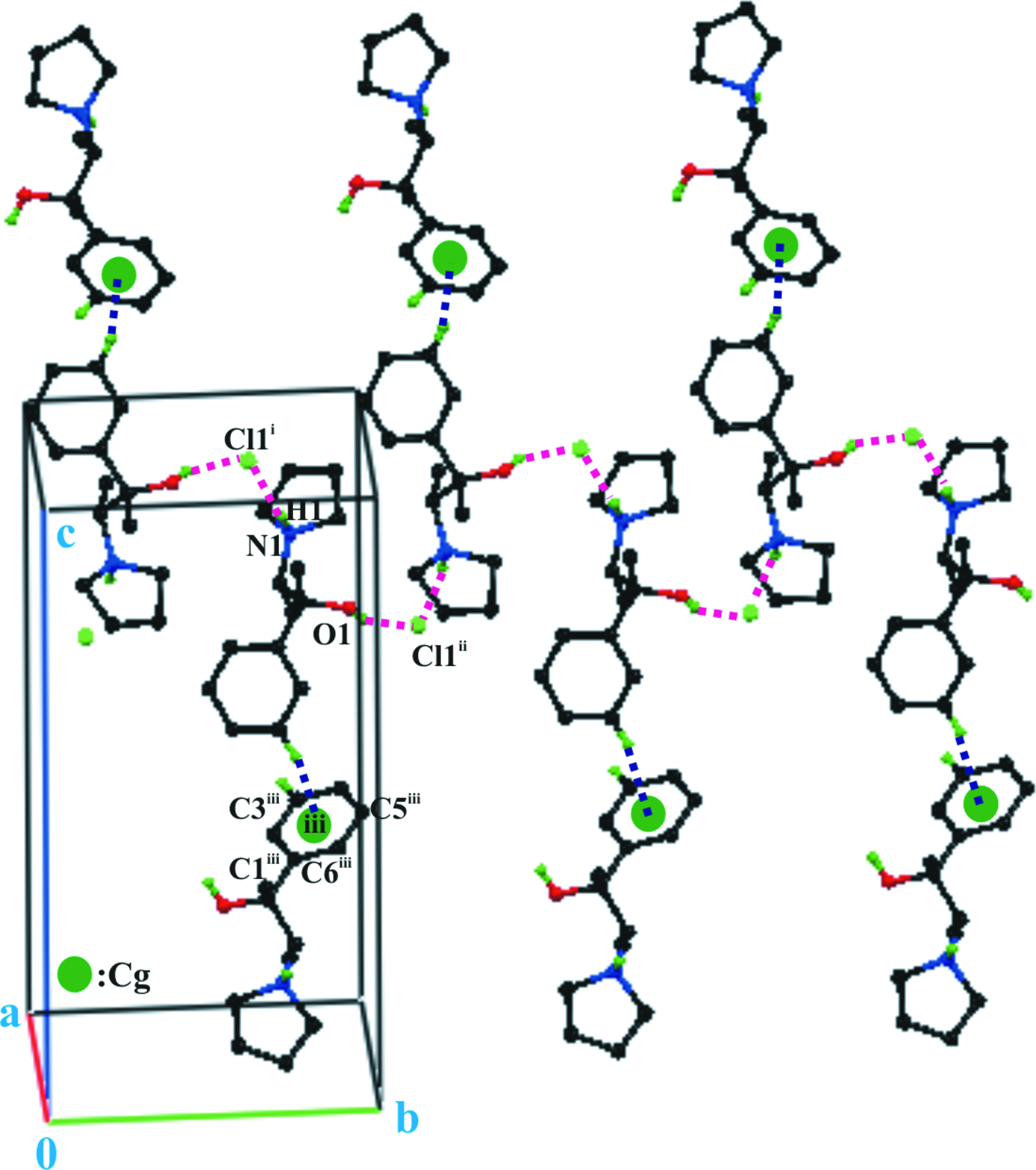

Supplement: Supplementary file 5 [file e-71-0o758-fig2.tif]
